# Reducing dimensionality for prediction of genome-wide breeding values

**DOI:** 10.1186/1297-9686-41-29

**Published:** 2009-03-18

**Authors:** Trygve R Solberg, Anna K Sonesson, John A Woolliams, Theo HE Meuwissen

**Affiliations:** 1 Norwegian University of Life Sciences, Department of Animal and Aquacultural Sciences, PO Box 5003, N-1432 Ås, Norway; 2 NOFIMA Marin, PO Box 5010, N-1432 Ås, Norway; 3 Roslin Institute (Edinburgh), Roslin, Midlothian, EH25 9PS, UK

## Abstract

Partial least square regression (PLSR) and principal component regression (PCR) are methods designed for situations where the number of predictors is larger than the number of records. The aim was to compare the accuracy of genome-wide breeding values (EBV) produced using PLSR and PCR with a Bayesian method, 'BayesB'. Marker densities of 1, 2, 4 and 8 N_e _markers/Morgan were evaluated when the effective population size (N_e_) was 100. The correlation between true breeding value and estimated breeding value increased with density from 0.611 to 0.681 and 0.604 to 0.658 using PLSR and PCR respectively, with an overall advantage to PLSR of 0.016 (s.e = 0.008). Both methods gave a lower accuracy compared to the 'BayesB', for which accuracy increased from 0.690 to 0.860. PLSR and PCR appeared less responsive to increased marker density with the advantage of 'BayesB' increasing by 17% from a marker density of 1 to 8N_e_/M. PCR and PLSR showed greater bias than 'BayesB' in predicting breeding values at all densities. Although, the PLSR and PCR were computationally faster and simpler, these advantages do not outweigh the reduction in accuracy, and there is a benefit in obtaining relevant prior information from the distribution of gene effects.

## Introduction

Approaches to the use of data from molecular markers in genetic evaluation for predicting breeding values have undergone considerable development as dense genome-wide marker technologies, such as high-density, high-throughput SNP chips, have become available. Currently, considerable attention is being paid to genomic selection with the approach of predicting genome-wide breeding values. Studies have demonstrated that the potential accuracies from dense molecular information are impressive, *e.g. *[[1-6], and [7]]. For example, [7] showed that it was possible to predict breeding values of unrecorded offspring using genomic selection with accuracies of 0.86 with only a small bias, for a trait with heritability 0.5, 1000 phenotypes and an effective population size of N_e _= 100. Whilst in general, the accuracies of evaluation will depend on a number of factors, one issue related to implementation is the computational demand. In [7], a Bayesian approach, 'BayesB' was used, which was computationally time-consuming and required some prior assumptions to be made concerning the potential number of QTL segregating and the prior distributions for QTL and marker effects.

This increase in the scale of molecular information results in data where, typically, the number of predictors (markers) is larger than the number of records (phenotypes). This statistical problem has been considered before, and several methods based on the multivariate regression theory, such as partial least square regression, (PLSR) and principal component regression (PCR) have been used for such situations. Both these techniques reduce the dimensionality of the set of regression variables by finding a small number of linear combinations of the original predictors, but the strategy for finding the linear combinations differ between the two methods. The regression methods have found fields of application primarily in chemometrics, econometrics and social sciences *e.g. *[8,9], but there have been only very few studies using PLSR and PCR concerned with their suitability for prediction of breeding values using large-scale molecular data, *e.g. *[10,11].

Therefore, one option for reducing the computational burden of 'BayesB' and for avoiding the use of prior distribution for marker effects is to make use of the simpler and faster PLSR and PCR algorithms. However, these algorithms have not been tested sufficiently in the context of genome-wide breeding value estimation, *e.g. *against 'BayesB' results, to decide upon their desirability of use. The study tested the hypothesis that an effective evaluation using genome-wide molecular data could be obtained using regression models of reduced dimensionality. Both PLSR and PCR were compared to the 'BayesB' for their accuracy and bias in predicting genome-wide breeding values.

## Methods

### Population structure and genome

#### Population structure

The simulation model was described in detail in an earlier paper [7]. Briefly, a population with an effective population size of N_e _= 100 was simulated over 1000 generations of random selection and mating with its genome subject to mutation. In generation *t *= 1001, the number of animals was increased to 1000 animals by factorial mating of 50 sires (i = 1–50) and 50 dams (i = 51–100) from generation 1000. The factorial mating was achieved by mating sire 1 to dams 51–70, sire 2 to dams 52–71, sire 3 to dams 53–72 and so on, and each dam had one offspring per sire. Animals in generation *t *= 1001 had 1000 offspring in generation *t *= 1002, produced by random mating among the parents in generation *t *= 1001. Animals in both generation *t *= 1001 and *t *= 1002 were genotyped for SNP markers.

#### Simulated genome

The size and structure of the genome were the same as described in [7] so that a direct comparison of the results was possible. The genome was simulated with 10 chromosomes each with a length of 100 cM each. Four density schemes of 1, 2, 4 and 8 markers/cM was evaluated, resulting in a total number of 1010, 2020, 4040 and 8080 markers across the 10 Morgan (M) genome. This would correspond to approximately 4000 to 32000 SNP markers in the Atlantic salmon (*Salmo salar*) genome, assuming a 40 M genome, or 3000 to 24000 SNP in the cattle genome, assuming a 30 M genome, respectively . However in this paper, densities will be scaled by the N_e _used to generate the markers, which was N_e _= 100 here, unless stated otherwise. This is because the linkage disequilibrium between markers is a function of 4N_e_c, where c is the distance between the markers and N_e _represents the marker density. Thus, the densities correspond to 1, 2, 4, and 8N_e_/M and will be expressed in this way throughout the paper.

The mutation rate of the markers was assumed to be 2.5 × 10^-5 ^per locus per meiosis and with this mutation rate, 99% of the potential markers were segregating at *t *= 1001. Markers with more than two alleles segregating at *t *= 1001 were converted to SNP as described in [7] so that the allele frequencies were as close as possible to 0.5. The typical distribution of the minor allele frequencies of the SNP markers at *t *= 1001 resembled a uniform distribution with an over-representation of markers with intermediate frequencies, which reflected the selection of the most informative markers that is undertaken in practice.

**Figure 1:**
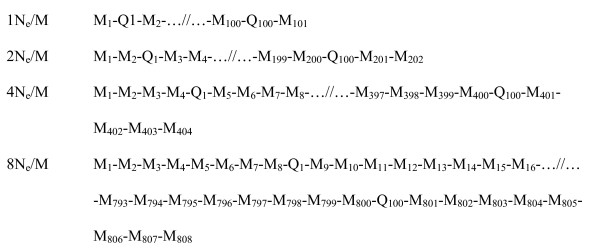
**Position of marker and QTL on each chromosome**. M_1_, M_2_, ...M_x _indicate the marker position, Q_1_, Q_2_, ...Q_100 _indicate the QTL position. The number of markers varied from 1N_e_/M (101 markers per chromosome) to 8N_e_/M (808 markers per chromosome). The number of QTL was kept constant at 100 per chromosome.

The potential number of QTL was kept at 100 per chromosome, distributed evenly over each chromosome (see Fig. 1). The actual number of segregating QTL at *t *= 1001 depended on the mutation rate which was assumed to be 2.5 × 10^-3 ^per locus per meiosis and resulted in the number of segregating QTL being typically 5 to 6% of the potential number with 93% biallelic. The distribution of the QTL allele frequencies of the positive QTL resembled a U-shaped distribution. The effects of a mutational allele of the QTL were sampled from the gamma distribution with the shape parameter of 1.66 and scale parameter of 0.4 [12] with an equal probability of a positive or negative effect.

The linkage disequilibrium (LD) that is generated by this population structure is described in [7]. The r-squared value increased when the marker density increased, and followed the expected value of r-squared well when allowing for mutations. Since the r-squared estimates were close to their expected values, the population was assumed to be close to a state of recombination-drift balance.

#### Phenotypic values

Phenotypic values for animals were first generated in generation *t *= 1001, and simulated as:

**P**_i _= **TBV**_i _+ **ε**_i_, where **TBV**_i _was the true breeding value for the *i*'th animal and **ε **~ N(0, σ^2^_e_). The variance of the additive genetic effects (σ^2^_a_) varied somewhat from replicate to replicate, but was on average 1 (s.e = 0.118). The environmental variance (σ^2^_e_) was kept constant and equalled 1. Hence, the heritability varied between replicates, but was on average 0.5 (s.e = 0.026) for all 20 replicates calculated from the 1N_e_/M scheme.

### Methods for estimating breeding values

Three methods for estimating breeding values were compared on each replicated dataset: PLSR, PCR and 'BayesB'. The basic idea of PLSR and PCR is to reduce the number of predictors with a smaller number of linear combinations of the predictors, with the additional property of pair-wise independence within the set of the constructed variables. From here on, the term latent variables will be used for these combinations of predictors applied to PLSR, while the term principal components will be used for PCR. The main difference between PLSR and PCR is in the method for constructing the latent variables or principal components. PLSR maximises the amount of covariance between the standardized predictors and response for a given number of latent variables, so that the covariance between the set of latent variables and phenotypes is as high as possible. In contrast, PCR maximises the proportion of total variance among the original predictors explained by the set of principal components. The third method, 'BayesB' makes prior assumptions on the amount of genetic variance and the distribution of gene effects, and breeding values are estimated from the data points by Bayesian methods.

#### Principal component regression (PCR)

For PCR, the principal components associated with the largest eigenvalues of the **X**'**X **matrix were extracted and used to predict the **y **values. The following steps were performed with PCR, when fitting c principal components:

1. Marker genotype data was organised, as *p *× *m *matrix (**X**), where *p *is the number of phenotypic records (1000 animals in this case), *m *is the number of marker genotypes. Genotypes were scored as 1 for genotype AA, 0 for heterozygote (Aa or aA) and -1 for aa. Hence the size of the **X **matrix varied from 1000 × 1010 (for 1010 markers) to 1000 × 8080 (for 8080 markers), with each column containing the set of genotypes for a single marker.

2. The marker genotype matrix **X **and **y **were standardised such that each column had a mean of zero and standard deviation of 1.

3. Singular value decomposition was then performed on the **X **matrix to find the principal components, and the *c *first components enter as columns in the matrix **U **[13].

4. The regression coefficients were obtained as **b**_PCR _= **US**^-1^**U**', where **S**^-1 ^is a diagonal matrix of the *c *highest singular values obtained from step 3.

5. Calculation of estimated breeding values (EBVs) was performed as explained in section 2.3.

The correlation between TBV and EBV was calculated when *c *= 10, 50, 100, 150, etc. components were fitted. The number of principal components that gave the highest correlation between TBVs and EBVs was used for each density.

#### Partial least square regression (PLSR)

In PLSR, the latent variables are constructed whilst accounting for their relationship to the data **y**, *i.e. *the latent variables are the combinations of the **X **variables that maximise the covariance with **y**. PLSR reduces the dimension of the regression **y **= **Xb **+ **e**, where **X **is a *p *× *m *design matrix, and **y **is a *p *× 1 data vector by performing the regression **y **= **Tq **+ **e**, where **T **is a *p *× *c *vector of 'scores', **q **is a *c *× 1 vector of 'loadings', and generally *c *<<*m*. **T **is calculated as **XW**, where **W **is a matrix of weights. Column *h *of **T**, **t**_h_, is chosen to maximise the covariance with the data, and this is obtained by setting the corresponding weights column, **w**_h_, proportional to the 'deflated' **X**'**y**. The deflated **X**'**y **refers to that part of **X**'**y**, which is orthogonal to the earlier scores **t**_1_, .., **t**_h-1_. The **X**'**X **matrix was deflated similarly. The deflation of **X**'**X **requires the regression of **X **onto the scores **T**, *i.e. ***X **= **Tp **+ **f**, where **p **are the loadings from this regression, and **f **are the residuals. We used the SIMPLS algorithm [14], which for a single trait **y **vector becomes :

1. Phenotypic values and marker genotype data were pre-treated and standardized in the same way as described in section 2.2.1 for PCR.

2. set **a**_1 _= **X**'**y**; **M**_1 _= **X**'**X**; and **C**_1 _= **I**, then perform steps 3–8 for h = 1, ..., *c*:

3. **w**_h _= **a**_h_/sqrt(**a**_h_'**M**_h_**a**_h_), which are the weights for the **X **columns to obtain **t**_h _= **Xw**_h_. The **w**_h _are stored as columns in **W**.

4. **p**_h _= **M**_h_**w**_h_, which is the regression of **X **on **t**_h_. The **p**_h _are stored as columns in **P**.

5. **q**_h _= **a**_h_'**w**_h_, which is the regression of **y **on **t**_h_. Since **y **is a single trait, **q**_h _is a scalar and is stored in the column vector **q**.

6. **v**_h _= **C**_h_**p**_h_, standardised to have Euclidean length 1. The **v**_h _is that part of **p**_h_, which is orthogonal to the earlier **p**_1_, .., **p**_h-1 _vectors.

7. **C**_h+1 _= **C**_h_-**v**_h_**v**_h_', which spans the space orthogonal to the **p**_1_, .., **p**_h _vectors.

8. **a**_h+1 _= **C**_h+1_**a**_h_, which deflates the **a**_h _vector; and **M**_h+1 _= **M**_h_-**p**_h_**p**_h_', which deflates the **M**_h _matrix. Return to step 3.

The regression coefficients of PLS regression then become **b**_PLSR _= **Wq**. The correlation between TBV and EBV was calculated for *c *= 1, 2, 3, 4, 5, 7, 9, 12, 15 and 20 fitted latent variables. The number of latent variables, *c*, that maximised this correlation was used for each density.

#### 'BayesB'

The 'BayesB' algorithm is described in detail and was used in earlier papers [4,7]. The 'BayesB' model was used to estimate marker effects and is briefly described as **y **= μ**1**_p _+ Σ_i_**Z**_i_**g**_i _+ **e**, where **y **is the vector of phenotypes, **1**_p _is a vector of *p *ones, Σ_i _is the summation over all markers, **Z**_i _is a design matrix for the *i*'th marker, **g**_i _is the vector of marker effects and **e **is the error. The variance of the marker effects (σ^2^_*gi*_) was estimated for every marker using a relevant prior distribution, which was a mixture of an inverted chi-squared distribution and a discrete probability mass at σ^2^_gi _= 0. A Metropolis-Hastings algorithm was used to sample σ^2^_gi _from its distribution conditional on y*, *p*(σ^2^_gi _| y*), where y* denotes the data **y **corrected for the mean and all other genetic effects except the marker effect (**g**_i_) [15]. Given σ^2^_gi_, marker effects, **g**_i _was sampled from a Normal distribution as prior and using Gibbs sampling [16]. Estimated marker effects using 'BayesB' together with marker genotype of the animal was used to predict the breeding values, as explained in section 2.3.

### Prediction of breeding values and statistics

Breeding values for the *n *animals in generation *t *= 1002 were estimated using the SNP marker information and the phenotypes in generation *t *= 1001, and compared to the true breeding values (TBV) in generation *t *= 1002. The EBV of animal *j *for PLSR and PCR were obtained from:

EBV_*j *_= **X**_*j*_**b**_*a *_for *j *= 1...*n*

where **X**_*j *_denotes the *j*'th row of the **X **matrix corresponding to the set of genotypes for animal *j*, **b**_a _is the regression coefficient vector of method *a*, where *a *denotes PCR or PLSR, and is estimated from the data in generation *t *= 1001. For 'BayesB' the EBVs were calculated from:

$$\begin{array}{ccc} {\text{EBV}}_{\text{j}}=\sum_{\text{i}=1}^{\text{m}}{\text{Z}}_{\text{i}(\text{j})}{\hat{\text{g}}}_{\text{i}} & \text{for} & j=1...n \end{array}$$

where **Z**_*i*(*j*) _denotes the row of the **Z**_*i *_matrix corresponding to the genotype of animal *j *at locus *i*, and ${\hat{g}}_{i}$ is the estimate of the marker effects for locus *i*, estimated in generation *t *= 1001.

TBV were linearly regressed on EBV, where the regression coefficient reflects the bias of the breeding value estimates (a regression coefficient of one denotes unbiased estimates), and the correlation coefficient reflects the accuracy of predicting the breeding values.

## Results

### Number of principal components with PCR

**Figure 2:**
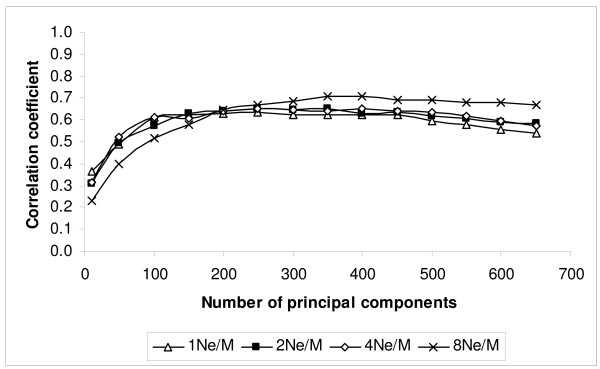
**Correlation coefficient between TBV and EBV using principal component regression (PCR) for different marker density schemes (1, 2, 4 and 8N_e_/M) when the number of principal components was varied**.

**Figure 3:**
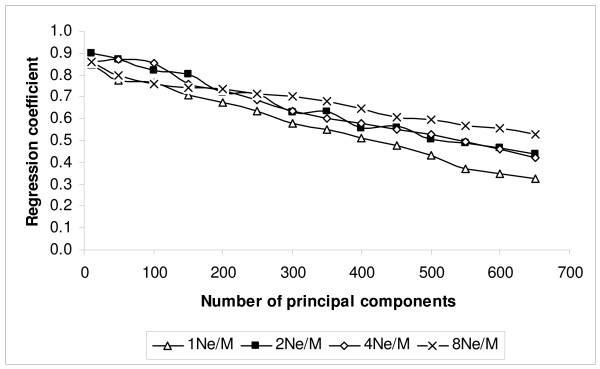
**Regression coefficient of TBV on EBV using principal component regression (PCR) for different marker density schemes (1, 2, 4 and 8N_e_/M) when the number of principal components was varied**.

Figures 2 and 3 show the correlation of TBV with EBV and the regression of TBV on EBV as a function of the number of principal components for PCR. For the three lowest marker densities, 1N_e_/M, 2N_e_/M and 4N_e_/M, the correlation reached a maximum when 250 principal components were fitted. For the 8N_e_/M marker density, the correlation reached a maximum when 350 principal components were fitted. After reaching the highest correlation between TBV and EBV, the correlation coefficient between TBV and EBV was approximately maintained until dropping more steeply when the number of principal components exceeded 400 (Fig. 2). The regression coefficient decreased almost linearly, and hence the bias increased, as more principal components were fitted (Fig. 3). In the following tables and comparisons, the results from fitting 250 principal components were chosen for 1N_e_/M, 2N_e_/M and 4N_e_/M marker density schemes, while 350 principal components were chosen for the 8N_e_/M marker density scheme, since this achieved the highest correlation between TBV and EBV with PCR.

### Number of latent variables with PLSR

**Figure 4:**
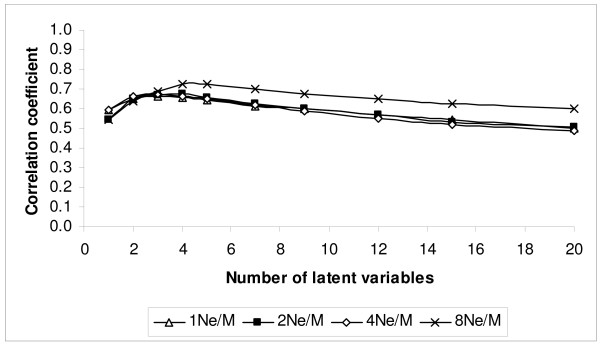
**Correlation coefficient between TBV and EBV using partial least square regression (PLSR) for different marker density schemes (1, 2, 4 and 8N_e_/M) when the number of latent variables was varied**.

**Figure 5:**
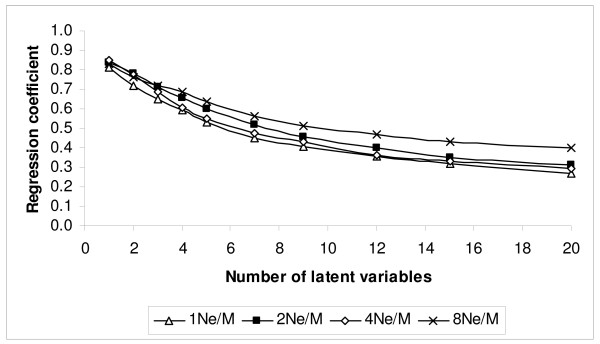
**Regression coefficient of TBV on EBV using partial least square regression (PLSR) for different marker density schemes (1, 2, 4 and 8N_e_/M) when the number of latent variables was varied**.

The correlation coefficient between TBV and EBV and the regression coefficient of TBV on EBV resulting from varying the number of latent variables from 1 to 20 are shown in Figures 4 and 5, respectively. Starting with one latent variable, the correlation coefficient between TBV and EBV increased until it reached a maximum between 2–4 latent variables (Fig. 4). The regression of TBV on EBV was close to 1 when only one latent variable was fitted, and dropped rapidly as more latent variables were added to the model (Fig. 5). In the following tables and comparisons the results from fitting two latent variables for the marker densities 1N_e_/M, 2N_e_/M and 4N_e_/M were chosen, while four latent variables were chosen for the 8N_e_/M marker density scheme, since this achieved the highest correlation between TBV and EBV with PLSR.

### Correlation

The correlation coefficients between TBV and EBV for the different marker densities and estimation methods together with their standard error are given in Table 1. The accuracy of estimating the breeding values increased as the density of the markers increased, as expected, since more information was available when more markers were fitted to the model. For PLSR, the correlation coefficient between TBV and EBV for the four densities (1, 2, 4 and 8N_e_/M) was 0.611, 0.655, 0.670 and 0.681, respectively. This was marginally greater than using PCR, which varied in a similar fashion from 0.604 to 0.665. Compared within densities the differences between PCR and PLSR were not significant, but across all densities PLSR gave a higher correlation than PCR by 0.016 (s.e = 0.008). The correlation coefficient between TBV and EBV for 'BayesB' was 8% greater than PLSR for the lowest marker density, and 18% greater for the highest marker density. Hence, the gap in accuracy between PLSR/PCR and 'BayesB' increased as the marker density increased.

**Table 1:** The mean correlation (r_TBV; EBV_) between TBV and EBV using principal component regression (PCR), partial least square regression (PLSR) and the 'BayesB' method for different marker densities, averaged over 20 replicates

	**PCR**	**PLSR**	**'BayesB'**
	
**Marker density**	**r_TBV; EBV _± s.e**	**r_TBV; EBV _± s.e**	**r_TBV; EBV _± s.e**
**1N_e_/M**	0.604 ± 0.012	0.611 ± 0.012	0.690 ± 0.036
**2N_e_/M**	0.639 ± 0.012	0.655 ± 0.012	0.790 ± 0.036
**4N_e_/M**	0.645 ± 0.012	0.670 ± 0.012	0.841 ± 0.036
**8N_e_/M**	0.665 ± 0.012	0.681 ± 0.012	0.860 ± 0.036

### Regression

The regression coefficients of TBV on EBV are summarized in Table 2. The most evident result is that this regression was higher for 'BayesB' compared to the regression methods, PLSR and PCR: the mean coefficient for 'BayesB' was > 0.87 for all marker densities, but was always < 0.76 for the regression methods, and this difference was large compared to the standard errors obtained. For 'BayesB' there was statistical evidence for a trend towards regression coefficients increasing towards 1 as marker densities increased. For the two regression methods, the pattern was more complex. More principal components and latent variables were fitted to optimise the correlations shown in Table 1 for 8N_e_/M than in scenarios with lower marker density, *i.e. *350 principal components and four latent variables were used for 8N_e_/M, while 250 principal components and two latent variables were used for PCR and PLSR respectively for the lower marker density schemes. Figures 3 and 5 show clearly that the regression coefficient decreases as the number of principal components or latent variables increase for both methods. With this caveat, at low densities (1, 2 and 4 N_e_/M) it appeared that the PLSR method resulted in greater regression coefficients than PCR (a difference of 0.07 with s.e = 0.01 over these densities), but this was reversed in favour of PCR (0.036 with s.e = 0.018) at 8N_e_/M. The regression coefficients for PCR appeared more stable compared to PLSR and exhibited a trend to greater regression coefficients as marker density increased.

**Table 2:** The mean regression coefficient (b_TBV; EBV_) between TBV on EBV using principal component regression (PCR), partial least square regression (PLSR) and the 'BayesB' method for different marker densities, averaged over 20 replicates.

	**PCR**	**PLSR**	**'BayesB'**
	
**Marker density**	**b_TBV; EBV _± s.e**	**b_TBV; EBV _± s.e**	**b_TBV; EBV _± s.e**
**1N_e_/M**	0.650 ± 0.012	0.758 ± 0.013	0.877 ± 0.013
**2N_e_/M**	0.683 ± 0.012	0.725 ± 0.013	0.879 ± 0.013
**4N_e_/M**	0.695 ± 0.012	0.754 ± 0.013	0.943 ± 0.013
**8N_e_/M**	0.691 ± 0.012	0.655 ± 0.013	0.923 ± 0.013

The standard errors (s.e) shown are derived from the pooled variance between replicates within each evaluation method.

### Computer time

Compared to the 'BayesB' method, the presented multivariate regression methods used much less computational time. The computer time for estimating the marker effects using the PCR, PLSR and 'BayesB' is presented in Table 3. The machine was an HP AlphaServer GS1280 with eight processors (EV7), of which only one processor was used at a time. PLSR used about 3 min per replicate to compute the marker effects for all marker densities, while PCR used somewhat longer time to calculate the marker effects, especially for higher marker densities, and the gap in computation time between PLSR and PCR increased as the marker density increased. However, the computation time for PLSR/PCR was very much reduced compared to the 'BayesB': *e.g. *'BayesB' used approximately 200 minutes to compute the marker effects for the lowest marker density, which was approximately 65 times longer than PLSR/PCR, and the computer time increased rapidly as the marker density increased (Table 3).

**Table 3:** Computation time for estimating the marker effects using principal component regression (PCR), partial least square regression (PLSR) and the 'BayesB' method

**Marker density**	**PCR**	**PLSR**	**'BayesB'**
**1N_e_/M**	~3 min	~3 min	~200 min
**2N_e_/M**	~15 min	~3 min	~700 min
**4N_e_/M**	~30 min	~3 min	~1600 min
**8N_e_/M**	~60 min	~3 min	> 2800 min

## Discussion

Two multivariate regression methods that reduce the dimensionality of the marker data were compared to a Bayesian method for the prediction of genome-wide breeding values based on SNP marker information and phenotypic records. In general, our results showed that it was possible to predict breeding values in our simulated genome using both multivariate regression methods, but the correlation between TBV and EBV were both reduced compared to those of 'BayesB'. The correlation between TBV and EBV increased as the marker density increased, because more information was available for predicting QTL genotypes, but most notably for 'BayesB'. The correlation is the accuracy of predicting EBV, whilst the regression indicates bias, and these correspondences will be used throughout the rest of the discussion. Hence, the results indicate that the regression methods deliver a lower accuracy and greater bias in predicting breeding values than 'BayesB', and are less responsive to the addition of further marker information.

The greater responsiveness to marker density of 'BayesB' was marked. For PLSR and PCR, the accuracy increased by 7% and 6% respectively from the lowest marker density (1N_e_/M) to the highest marker density (8N_e_/M), whilst in contrast 'BayesB' was 17% more accurate for the highest marker density compared to the lowest density. Hence, the gap in accuracy between PLSR/PCR and 'BayesB' increased as the marker density increased. From this result, it seems that the use of relevant prior information, as in the 'BayesB' method, was more valuable as the marker density increased.

Whilst the accuracy of prediction may be the primary parameter of interest, the regression of the TBV on EBV is relevant since it determines the bias in predicting genetic progress. One possible consequence is that this will contribute to decreasing the accuracy in predicting breeding values if the population used for providing estimates of breeding values spans more than a single generation of selection. In this attribute, as in accuracy, the advantage appears to lie in 'BayesB', with regression coefficients both closer to one than PLSR and PCR and increasing as marker density increased. Although, these biases may be corrected for by scaling the EBV such that Var(EBV) = Cov(EBV, TBV), and thus are not a major hindrance for the use of PLSR or PCR. The regression methods had increased bias as density increased because more principal components or latent variables were required to optimise the accuracy. Any use of PLSR or PCR would require optimisation on the number of principal components or latent variables, perhaps through cross-validation for each practical data set, although both accuracy and bias will depend on the number of phenotypes.

The main advantages using PLSR and PCR compared to the 'BayesB' method were the computing time and avoidance of the assumptions about prior distribution of marker effects made in the 'BayesB' model. PLSR and PCR were computationally much faster and simpler compared to the 'BayesB' method, *e.g. *the computation time for estimating the marker effects using PLSR was approximately 65 times faster than 'BayesB' for the lowest marker density. The gap in computation time, hence the computational costs, were increased for higher marker density. For example, the expected linkage disequilibrium (LD) for the same recombination rate will be reduced by doubling the effective population size N_e_. Hence, assuming the accuracy is primarily determined by the amount of LD, then a doubling of the number of markers is needed to achieve the same LD, a finding supported by [7]. Doubling the number of markers will double or triple the computation time needed, which is especially time consuming for 'BayesB'. Compared to PLSR and PCR, 'BayesB' has a greater potential for exploiting parallel computing, which was not used in this study, therefore the relative computational benefits of PLSR and PCR will diminish as parallel processing becomes cheaper and more common. This parallel computing implementation of BayesB will be highly needed because the number of markers is expected to increase for most species to 50 – 500 thousand.

Meuwissen *et al*. [4] used microsatellites at 1 N_e_/M density to compare least square regression after screening for significant QTL, BLUP and 'BayesB' for predicting genome-wide breeding values, and found accuracies of 0.318, 0.732 and 0.848, respectively. Solberg *et al. *[7] determined that SNP densities of 2- to 3-fold greater densities were required to achieve comparable accuracies. Therefore, an appropriate comparison may be made with the results of [4] with SNP at a density of 4N_e_/M in our study. For this density, PLSR and PCR had accuracies of 0.670 and 0.645. Hence, these results indicate that the Bayesian method 'BayesB' gave the highest accuracy, followed by BLUP, PLSR and PCR, and least square analysis combined with screening had the lowest accuracy.

A somewhat high heritability was used in this simulation study, therefore a comparison of the three methods BayesB, PLSR and PCR were additionally evaluated for a lower heritability of 0.25 (Table 4). For the highest marker density, the selection accuracy was reduced by 7% for the BayesB method, and 16% for the two regression methods. For the lowest marker density, the selection accuracy was reduced by 14% for the regression methods and 12% for the BayesB method. No significant differences were observed between the PLSR and PCR. Even if the selection accuracy was reduced in all cases, the same "ranking" of the methods remain, namely, BayesB performed better than PLSR and PCR.

**Table 4:** Comparison of the three methods for the lowest (1Ne/M) and the highest (8Ne/M) marker density, when the heritability was 0.25

	**PCR**	**PLSR**	**'BayesB'**
	
**Marker density**	r_TBV; EBV _± s.e	r_TBV; EBV _± s.e	r_TBV; EBV _± s.e
**1N_e_/M**	0.452 ± 0.009	0.465 ± 0.011	0.566 ± 0.018

**8N_e_/M**	0.510 ± 0.012	0.504 ± 0.014	0.793 ± 0.018

A conclusion from this study is that if some relevant information is known *a priori *then methods that utilize relevant prior information will be more accurate. The 'BayesB' method assumed a mixture of distributions of an inverted chi-square with a discrete probability mass at zero as the relevant prior distribution of marker effects, to model an increase in the number of markers with an effect of zero. The simulated QTL effects followed a gamma distribution with a shape parameter of 1.66 and a scale parameter of 0.4 [12] with equal probability of positive or negative effects. In practice, we do not know the exact distribution of the QTL effects. Although the distribution used for simulating the QTL effects and that used for analysing the data did not agree exactly, 'BayesB' approximates the prior distribution of the QTL effects better than the regression methods. From a Bayesian perspective, PLSR and PCR might be viewed as representing a limiting form where the prior distribution for regression coefficients is normally distributed with an increasingly large variance. This closer correspondence between the prior used for evaluation and the simulated distribution of QTL perhaps explains in part the higher accuracies obtained with 'BayesB'.

PLSR and PCR give an alternative solution to 'BayesB' to estimate marker effects. They provide a rapid analysis of large amounts of data to obtain EBVs from high-density markers. The only assumptions are the additivity of marker effects, and that few linear combinations of markers can explain most variability in the data. However, whilst this simulation study showed that reducing the dimensionality of the data gave a reasonably high accuracy of selection, the accuracy was less than that obtained from 'BayesB', and this difference increased with increasing marker density. To obtain full benefits of genome-wide selection, use of relevant *a priori *information about the distribution of the QTL effects is preferable, since genotyping costs are very high relative to computational costs. These relevant prior distributions need to be obtained by acquiring greater knowledge of the genomic architecture.

## Competing interests

The authors declare that they have no competing interests.

## Authors' contributions

TRS simulated the datasets, carried out the analysis and drafted the manuscript. TM helped to carry out the study and drafting the manuscript. All authors have read and approved the final manuscript.
